# Benzodiazepine-Induced Acute Psychosis Leading to Self-Enucleation and Auto-Cannibalism

**DOI:** 10.7759/cureus.92000

**Published:** 2025-09-10

**Authors:** William Shyy, Michael D Yashar

**Affiliations:** 1 Emergency Medicine, University of California Los Angeles, David Geffen School of Medicine, Los Angeles, USA; 2 Medicine, University of California Los Angeles, David Geffen School of Medicine, Los Angeles, USA

**Keywords:** agitated delirium, auto-cannibalism, autoenucleation, benzodiazepine use, clinical case report, drug-induced psychosis, oedipism, sedation delirium, self-enucleation, self-mutilation

## Abstract

Lorazepam is a benzodiazepine that has been FDA-approved as a fast-acting anxiolytic and sedative. It is one of the most commonly used medications for these indications and is considered highly effective and safe. We present a case of a patient without prior mental illness who received intravenous lorazepam to treat anxiety and claustrophobia in order to facilitate an MRI, and who subsequently developed acute psychosis, performed self-inflicted digital enucleation of his left eye, and then ingested it. Despite an extensive evaluation by ophthalmology, neurology, neurosurgery, infectious disease, and psychiatry, the ultimate etiology of the patient’s psychosis was thought to be acute delirium due to benzodiazepine use. This case report demonstrates a very rare but life-threatening paradoxical side effect of benzodiazepines, as well as the extensive evaluation patients should undergo for acute psychosis in the hospital. Recognition and awareness of this paradoxical drug side effect are essential for providers administering benzodiazepines to ensure patient safety.

## Introduction

Benzodiazepines have been widely used for a variety of clinical indications since the first benzodiazepine drug, chlordiazepoxide, was created in 1955. Benzodiazepines act by binding to GABA (gamma-aminobutyric acid) receptors in the central nervous system, enhancing the inhibitory effects of GABA, leading to anxiolytic and sedative effects [1,2]. Lorazepam is a common benzodiazepine with a prolonged elimination half-life ranging from 14 to 18 hours and is FDA-approved to treat anxiety disorders, anxiety-related insomnia, anesthesia premedication, and status epilepticus [3,4]. It also has off-label uses for rapid sedation of an agitated patient, alcohol withdrawal, panic disorder, delirium, catatonia, vertigo, and chemotherapy-associated nausea and vomiting.

While benzodiazepines have been in clinical use for 70 years, case reports also document rare incidents of paradoxical reactions characterized by agitation and rage since the 1960s [5-7]. The etiology of these drug-induced psychoses and aggressive outbursts has not been well understood, but such paradoxical reactions have occurred in patients of diverse demographics with and without a prior history of mental illness. Cases have varied from those of initially aggressive patients whose behavior worsened after being treated with benzodiazepines to others without any significant mental illness history who developed extreme aggression and hostility after treatment with benzodiazepines [5-7]. These presentations have been found to range in character, with variable responses including anxiety, anger, violence, impulsivity, mania, and self-injurious behaviors. Given the rare incidence of this side effect and the inability to predict who may experience an abrupt and unexpected paradoxical reaction, early recognition of this potential adverse drug reaction, along with a thorough evaluation of other etiologies for such acute behavioral changes, is essential.

## Case presentation

A 62-year-old male with a past medical history of hepatitis C, remote substance use disorder, brain tumor status post resection complicated by lateral brainstem infarction, status post ventriculoperitoneal (VP) shunt, and C3-C6 laminectomy/posterior fusion two weeks prior to presentation was seen in the emergency department (ED) with ongoing neck pain since his recent surgery, as well as headache upon standing for the prior 24 hours. The patient endorsed stable bilateral arm weakness since his spine surgery, but no worsening of symptoms. He also described experiencing positional headaches for the past few months after undergoing a VP shunt revision, which had become much worse since the night prior. He reported headaches upon sitting up or standing, with improvement when supine. He had been taking oxycodone for pain but expressed concern that his acute rehabilitation facility was not administering his pain medications on time and his pain was inadequately treated. The patient otherwise denied fever, chills, vision changes, new weakness or numbness, or trauma. The patient’s vital signs were within normal limits, without fever. His physical exam was unremarkable except for a chronic left facial droop with left ptosis, a clean-appearing cervical spine surgical incision, and mild proximal upper extremity weakness limited by severe neck pain, without hyperreflexia. The patient endorsed neck pain, though he appeared comfortable overall, was alert and oriented to person, place, and time, had clear and linear speech, a calm affect, and exhibited appropriate insight and judgment regarding his post-surgical pain. He stated that he had requested to come to the emergency department to have his neck pain and headache evaluated.

**Figure 1:**
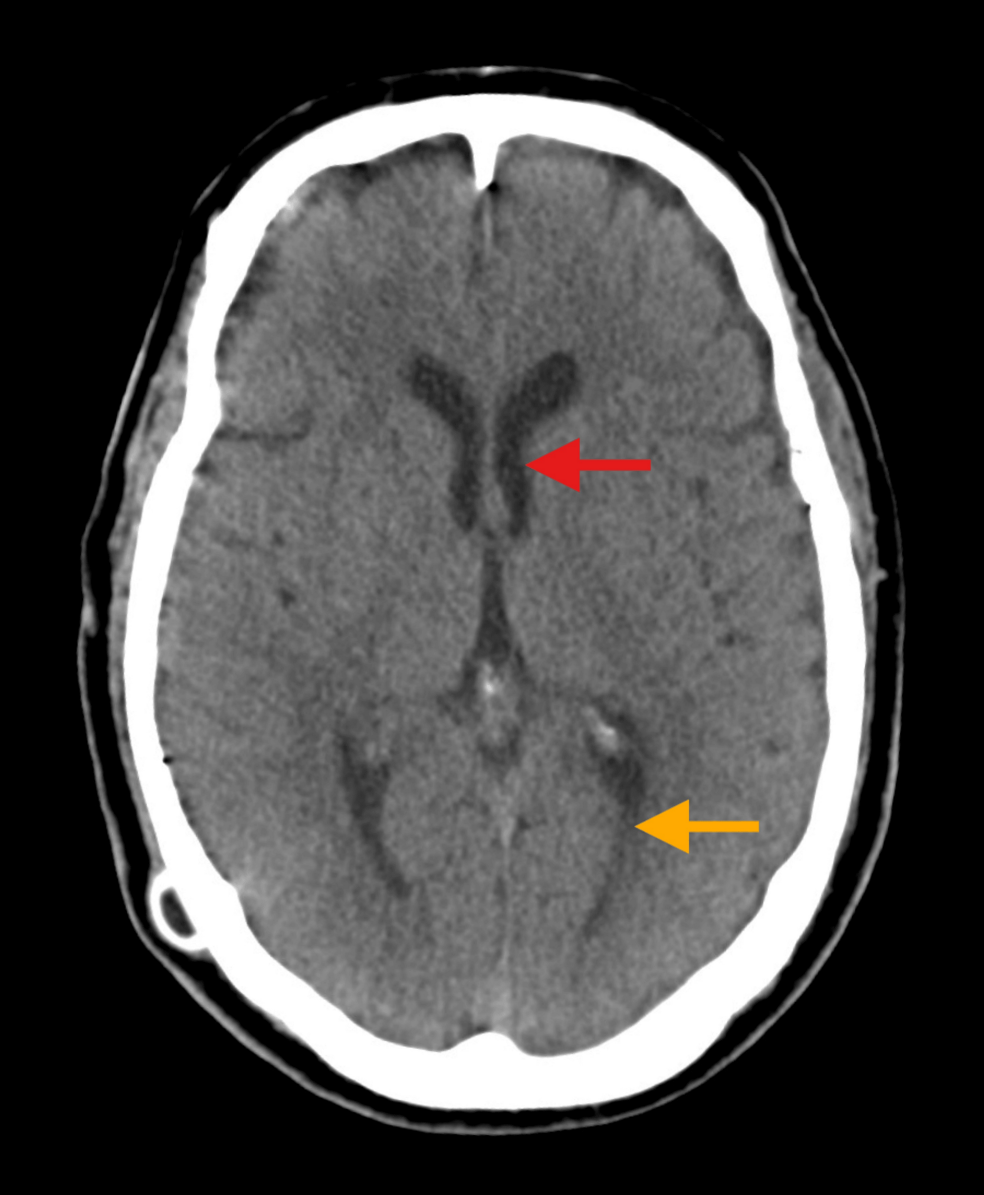
CT brain without contrast Decreased ventricular caliber of the lateral horns: anterior (red arrow) and posterior (orange arrow).

The emergency medicine (EM) provider ordered a complete blood count and basic metabolic panel, which were normal, and a CT brain and cervical spine without contrast, which showed no acute findings aside from a decreased ventricular caliber compared to imaging prior to his VP shunt revision (Figure 1).

**Figure 2:**
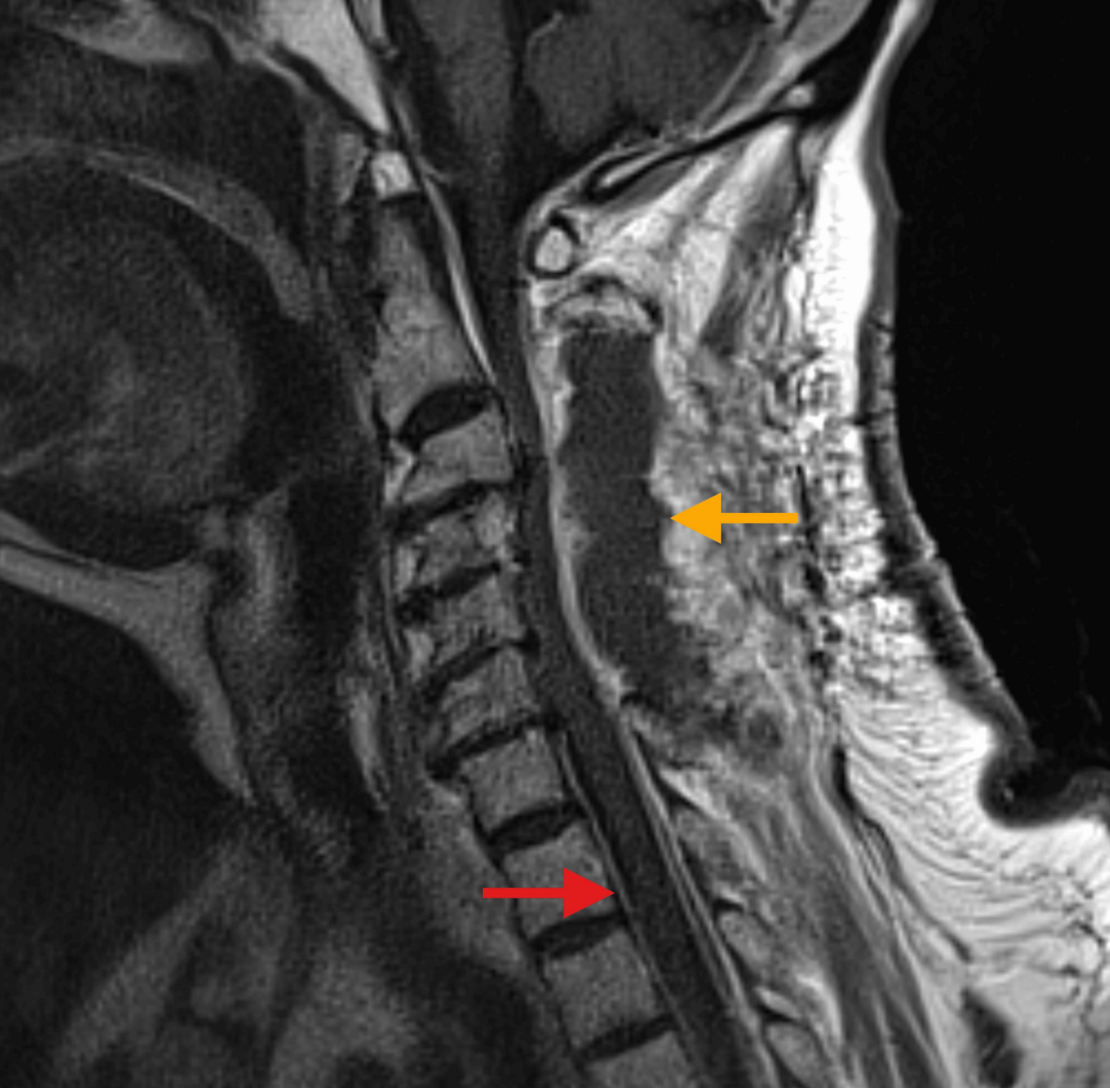
MRI cervical spine with contrast, T1-weighted Dural enhancement of the cervical spinal canal (red arrow) and dorsal epidural non-enhancing fluid collection (orange arrow).

Neurosurgery was consulted and recommended an MRI of the cervical spine with and without contrast to rule out a cerebrospinal fluid leak. The patient was given three doses of 1 mg intravenous (IV) hydromorphone over a 10-hour period for pain management, and 1 mg IV lorazepam at 6 p.m. prior to his MRI for claustrophobia. The MRI showed dural enhancement concerning for intracranial hypotension, as well as a dorsal epidural non-enhancing fluid collection (Figure 2).

Neurosurgery evaluated the patient, who felt he was safe to be discharged with outpatient neurosurgery clinic follow-up to discuss consideration of a potential VP shunt revision, as they thought the patient’s headache was due to over-shunting. The patient subsequently declined to return to his skilled nursing facility after being counseled that there was no indication for inpatient hospitalization, and so social work and case management were consulted. A plan was developed to observe the patient in the emergency department overnight and discuss his concerns with case management in the morning, as the patient may have been a candidate for more immediate placement in a higher-level-of-care facility pending insurance authorization.

The patient unexpectedly became agitated around 2 a.m., yelling, removing cardiac monitor leads, and demonstrating non-compliance with his cervical collar. The patient’s nurse responded and was able to de-escalate him verbally. According to the nurse’s documentation from this interaction, the patient “said he did not know why he was yelling but he just wanted to.”

Shortly afterward, at 3:30 a.m., the patient’s nurse noted he had a bloody face with apparent lacerations to the left eye and left nose, with bloody hands and pieces of flesh on his chest, neck, and face. The EM provider then discovered the patient’s left globe was missing from his left orbit, and when asked if he had pulled the globe out, the patient stated “yes” and that “I ate it,” while expressing suicidal intent. The room was thoroughly searched, and no globe remnants were ultimately found. The patient was placed in restraints, given 5 mg intramuscular (IM) olanzapine at 4 a.m., followed by 2 mg IV lorazepam at 7 a.m., and was then transferred to the main campus emergency department for higher-level-of-care evaluation by several specialty consulting services.

**Figure 3:**
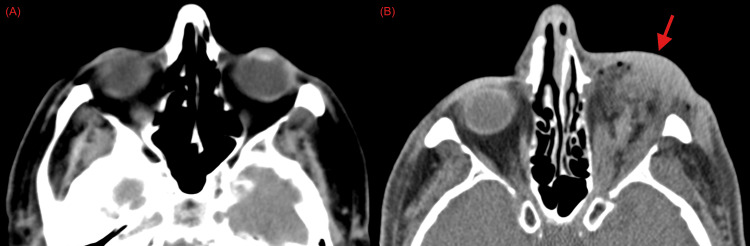
CT brain without contrast, including orbits (A) Initial imaging before self-enucleation. (B) Subsequent repeat imaging after self-enucleation with missing globe (red arrow).

On arrival at the main campus emergency department at 9:15 a.m., the patient was evidently agitated, yelling and kicking at staff, with mostly incomprehensible speech aside from threatening to “get his gun to shoot staff.” When asked why he had injured himself, the patient’s response was unintelligible. Upon his presentation at this time, the patient was immediately given 10 mg IM olanzapine for agitation. Shortly afterward, at 10:20 a.m., an additional 1 mg IV lorazepam was given, and he tolerated a repeat CT brain study, which confirmed self-enucleation (Figure 3).

The patient was then admitted to the internal medicine hospitalist service, with consults placed to neurosurgery, neurology, ophthalmology, psychiatry, and infectious disease. The consulting teams advised further evaluation, including an MRI of the brain, electroencephalogram (EEG), and lumbar puncture with cerebrospinal fluid testing for bacterial, fungal, and viral meningitis, including meningoencephalitis and autoimmune/paraneoplastic Mayo panels. They also recommended empiric treatment with broad-spectrum antimicrobials (vancomycin, cefepime, acyclovir) for infectious encephalopathy, olanzapine for psychosis, and empiric thiamine supplementation, each of which was initiated. All of the aforementioned laboratory tests, EEG, and imaging studies were within normal limits except for the previously noted MRI findings of the postoperative cervical spine fluid collection, which neurosurgery did not believe was concerning for a septic collection. While inpatient, the patient required four-point restraints for the first three days while initially remaining floridly and violently psychotic. His mental status gradually improved to alert and oriented to person, place, and time within approximately 48 hours, though he had persistent delusions that medical providers were trying to kill him and that he had been kidnapped and transferred to a boat. After seven days of hospitalization, his mental status had returned to baseline with resolution of his psychosis and delusions, and he acknowledged that he had pulled out his eye, but without recall of why he performed the self-enucleation. Methocarbamol was added to his oxycodone for improved pain control. Three days after methocarbamol was initiated, he had another acute psychotic episode during which he was re-evaluated by neurology, neurosurgery, psychiatry, and infectious disease, who felt this was a recurrent acute psychotic episode likely due to the methocarbamol. The methocarbamol was stopped, and his mental status again returned to normal over a few days.

Overall, this presentation was most consistent with acute psychosis due to lorazepam after an extensive work-up ruled out other causes such as infectious or inflammatory encephalopathy. Of note, this patient had no prior history of adult mental illness or psychiatric care and had been sober from drugs and alcohol for 15 years. Given the proximity of his acute psychotic episode to the preceding lorazepam administration, and his worsening psychosis after receiving several additional doses for his severe agitation, it was ultimately concluded that the patient had suffered a rare episode of drug-induced delirium leading to psychosis. Of further note, the patient had a recurrent episode of psychosis several days after he had returned to baseline, which was also thought to be due to drug-induced delirium from methocarbamol.

## Discussion

This case highlights the potential for extremely rare paradoxical reactions to benzodiazepines, which can lead to agitation and rage instead of the expected sedative and depressant effects. The differential diagnosis for acute agitation in a patient with recent spine surgery and a prior brain tumor status post resection and VP shunt placement should be broad and includes meningitis, encephalitis, stroke, postoperative surgical site infection, delirium, and new-onset psychiatric illness such as schizophrenia or bipolar disorder. After extensive diagnostic testing and repeated evaluation over a week by the internal medicine hospitalist, ophthalmology, neurosurgery, neurology, infectious disease, and psychiatry services, infectious, inflammatory, and neurovascular causes of the patient’s self-enucleation and auto-cannibalism were ruled out. After multispecialty consensus, where all potential causative factors were considered, including the timing of lorazepam administration and the medication’s known lengthy elimination half-life, it was felt that the patient had most likely suffered a paradoxical reaction to benzodiazepines causing progressive and severe psychomotor agitation. Due to the patient’s overall displeasure with his care at his skilled nursing facility, he had an extreme reaction when told he would be sent back, leading to the impulsive and irrational self-harm act, which was associated with an acute psychotic episode spanning almost a week. It is important to note this happened in a patient with no prior history of adult mental health issues, no anger or behavioral problems per family and friends, and no prior history of self-harm or encephalopathy.

Lorazepam has a relatively simple mechanism of action: to enhance the inhibitory actions of GABA in the central nervous system to reduce anxiety, aggression, alcohol withdrawal, and seizure activity. It remains unclear why a very small minority of patients given a benzodiazepine will experience a paradoxical reaction, regardless of the indication for its administration. One case report describes a patient who was given lorazepam for alcohol withdrawal and subsequently became agitated, defecated in a sink, and drank his own urine, returning to normal when lorazepam was held [8]. Another patient with a history of depression was given diazepam for anxiety and developed aggressive fantasies, physically abused her daughter, and repeatedly hit her head against walls; she also returned to normal when diazepam was stopped [9]. The severity of paradoxical reactions varies greatly and unpredictably, from increased anxiety and insomnia to overt physical violence [10]. Other patient case reports also describe instances of self-enucleation and subsequent auto-cannibalism thought to be secondary to underlying primary psychiatric conditions and/or recreational drug use; however, none of these occurred in the setting of benzodiazepine or other prescribed medication administration [11-14].

## Conclusions

Agitation and behavioral disturbances should be considered a rare but well-documented paradoxical phenomenon of benzodiazepine administration. Severe behavioral disturbances after benzodiazepine administration should be anticipated, recognized early, and immediately managed to avoid risks of patient self-harm, while concurrently evaluating and treating other potential etiologies. We hope that early identification of this rare, though life-threatening, paradoxical reaction will improve patient safety by assisting healthcare providers in preventing the administration of further benzodiazepines, which may carry potentially deleterious and unintended consequences.
